# Inter-observer variability influences the Lugano classification when restaging lymphoma

**DOI:** 10.4102/sajr.v22i1.1357

**Published:** 2018-07-31

**Authors:** Jacobus Möller, Tiaan Steyn, Nantes Combrinck, Gina Joubert, Alicia Sherriff, Jacques Janse van Rensburg

**Affiliations:** 1 Department of Clinical Imaging Sciences, Universitas Academic Hospital and University of the Free State, South Africa; 2 Department of Biostatistics, University of the Free State, South Africa; 3 Department of Oncology, Universitas Academic Hospital and University of the Free State, South Africa; 4 Department of Radiology, Universitas Academic Hospital and University of the Free State, South Africa

## Abstract

**Background:**

Lymphoma is an important and potentially curable oncological disease in South Africa. The staging and restaging of lymphoma have evolved over the years, with the latest international consensus guideline being the Lugano classification (LC). Prior to routine implementation of the LC, its robustness in the local setting should be determined.

**Objectives:**

To determine the Inter-observer variability in response assignment when applying the LC in patients with lymphoma who were staged and restaged with computed tomography. In case of excessive discordance, specific mitigating measures will have to be taken before and during any proposed implementation of the LC.

**Method:**

A total of 61 computed tomography scans in 21 patients were evaluated independently by four reviewers according to the LC, of which 21 scans were done at baseline, 21 at initial restaging and 19 at follow-up restaging. A retrospective comparative analysis was performed. Kappa values were calculated to determine agreement between observers.

**Results:**

Only a moderate inter-observer agreement of 52% in the overall response classification was demonstrated. The most important sources of discrepancy were inconsistency in the assessment of target lesion regression to normal, determining the percentage change in the summed cross-sectional area of the target lesions and ascribing new lesions as either due to lymphoma or other causes.

**Conclusion:**

Implementing the Lugano classification when restaging lymphoma is desirable to improve consistency and to conform to international guidelines. However, our study shows substantial inter-observer variability in response classification, potentially altering the treatment plan. Dedicated training and continuous quality control should, therefore, accompany the process.

## Introduction

South Africa has a high burden of cancer, of which lymphoma is an important and potentially curable condition.^1^ The oncological staging and restaging of patients with lymphoma have undergone several changes over the years, with the latest international consensus guideline being the Lugano classification (LC).^2,3^

**FIGURE 1:**
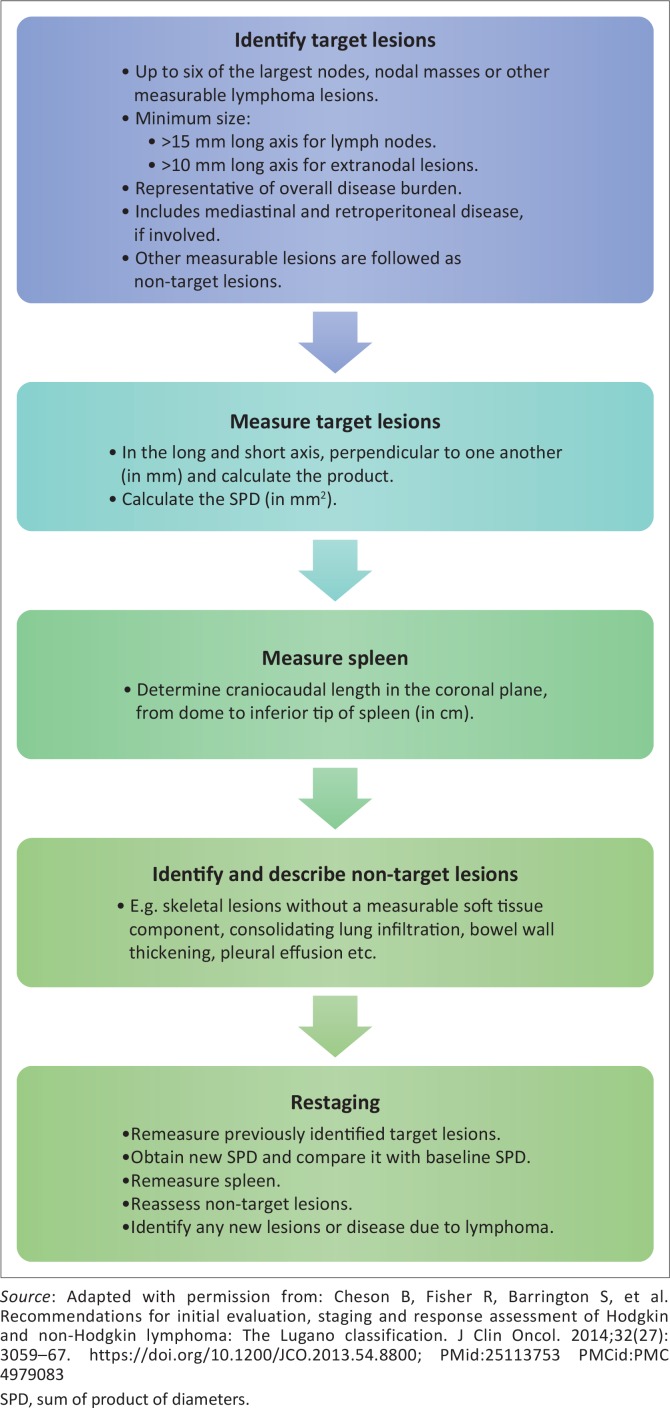
Applying the Lugano classification with computed tomography.

**FIGURE 2:**
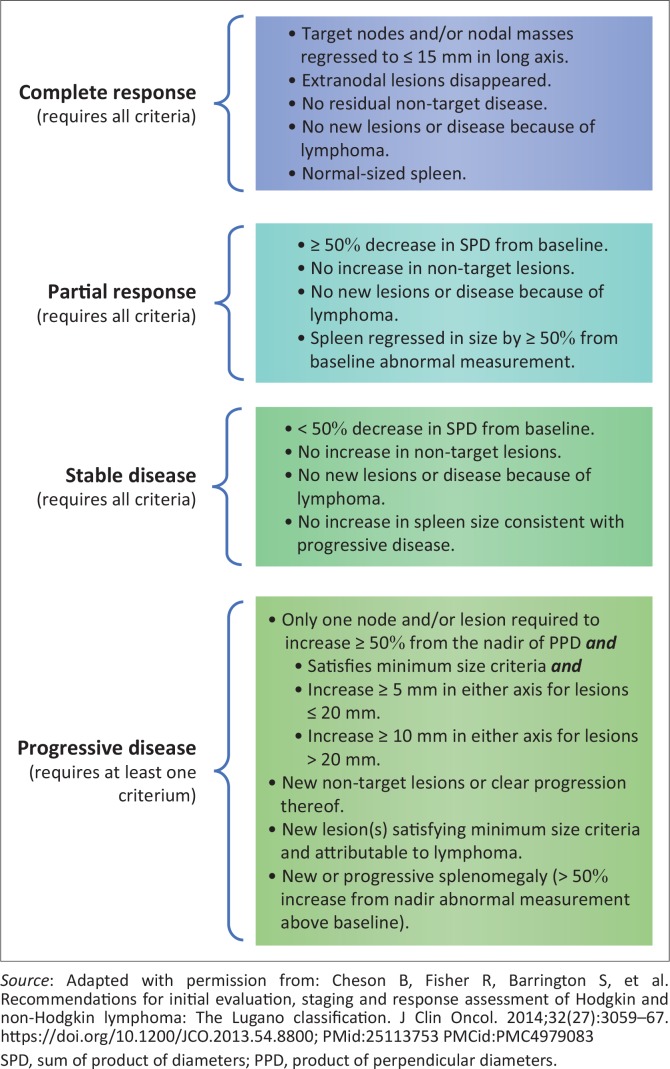
Response to treatment according to the Lugano classification.

The Lugano classification (LC) (Figures 1 and 2) was conceived as an unambiguous and universally applicable lymphoma staging system, suitable for both clinical practice and trials, to enable multicentre investigative studies and facilitate the evaluation of new therapies by healthcare authorities.^4^ Internationally, combined positron emission tomography and computed tomography (PET-CT) is considered to be the principal imaging method for staging and restaging of lymphoma, although stand-alone CT is regarded as an acceptable alternative when PET-CT is unavailable.^5,6^

However, the South African circumstances, in general, and those of the Free State province, in particular, differ significantly from the developed world.^7,8,9^ For instance, PET-CT is unavailable in government hospitals in the Free State and four other South African provinces, at least in part because of limited financial resources. The situation is complicated further by the large rural population, which hampers regular follow-ups at centralised treatment centres and the advanced nature of the lymphoma at presentation, often with concomitant human immunodeficiency virus (HIV) and opportunistic infections, such as tuberculosis (TB).^10^

Size measurement is a fundamental part of CT interpretation in both oncologic and non-oncologic settings.^11^ CT measurements are used as imaging biomarkers or surrogate endpoints for assessing treatment response when applying the LC with lymphoma. Patients are then assigned to one of the following categories, predominantly based on the measurements obtained with CT: complete response (CR), partial response (PR), stable disease (SD) or progressive disease (PD).^12,13^

Possible sources of error during CT analysis in lymphoma include intra- and inter-observer variability and inconsistent application of measurement criteria, which may lead to discordance in the evaluation of the tumour response.^14,15,16^ Furthermore, Ford et al.^17^ described a number of errors that can lead to misclassification of the treatment response: firstly, human error, where lesions are missed during evaluation, incorrectly measured or inappropriately regarded as target lesions; secondly, data errors, where all the available imaging and clinical information were not considered; thirdly, application errors, where the response criteria were inconsistently applied; and fourthly, conclusion errors, where the reader makes an incorrect assessment of the otherwise valid data.

To the best of our knowledge, there is no published research that specifically addresses inter-observer variability when applying the LC to CT-scans performed in patients with lymphoma. Studies in a similar manner either used different CT criteria (International Harmonization Project [IHP]), a different imaging modality (PET-CT), or assessed the CT response criteria utilised for solid tumours (response evaluation criteria in solid tumours [RECIST]).^18,19,20^

The inconsistent assignment of response categories among different reviewers has obvious negative implications for patient management. Assigning an erroneous response category will lead to either overtreatment or undertreatment, with its associated risks.^17^ The objective of this study was to determine the inter-observer reproducibility of the response to treatment assessment according to the LC when using CT in patients with lymphoma. This would demonstrate whether the LC is robust enough to be introduced directly into routine clinical practice in the South African public sector setting or whether additional measures, such as dedicated training and continuous quality control, would be necessary.

## Research methods and design

### Setting

Locally (Bloemfontein), Universitas hospital and its annexes serve as the oncology referral centre for central South Africa and Lesotho. Standard practice with lymphoma is to perform a baseline CT-scan prior to the initiation of therapy. Generally speaking, an initial restaging scan is performed after two to four cycles of chemotherapy with follow-up restaging scans performed after four to six cycles and at the completion of treatment. The result of the CT-scan is integrated with that of the bone marrow biopsy and clinical findings by the oncologist. Patients who do not at least have a PR to therapy after four cycles of chemotherapy are considered for second-line chemotherapy. Patients with radiological evidence of residual disease at the completion of treatment or interim PD may need histological confirmation, followed by consideration of salvage chemotherapy and/or radiotherapy.

### Patients and materials

Adult patients with newly diagnosed lymphomas, who presented for their baseline and restaging CT-scans at Universitas Academic Hospital Complex between January and November 2017, were considered for inclusion in the study. A total of 21 patients met the aforementioned criteria and were included in the study. A total of 61 CT-scans were performed in these patients: 21 scans were done at baseline, 21 scans at initial restaging and a further 19 scans at subsequent follow-up restaging. Nine patients had one follow-up restaging scan, while five patients had two follow-up restaging scans.

Most (54) of the scans were performed with General Electric’s six-slice scanner; the other seven scans were performed with General Electric’s 64-slice scanner. With the six-slice scanner, scans were acquired from the skull vertex to the pubic symphysis. Multiplanar reconstructions of the neck were performed at 1.25 mm slice thickness and for the rest of the body at 2.5 mm. With the 64-slice scanner, the base of the skull to the symphysis was included, and 1.25 mm slice thickness multiplanar reconstructions were performed of the whole body.

### Design, procedure and analysis

A retrospective, comparative analysis was performed. Four reviewers reviewed all the scans of every patient independently. Three of the reviewers were registrars, with three to five years of experience in interpreting body CT, while one reviewer was a specialist with more than 10 years of experience. The reviewers were provided with the clinical information that is routinely available in normal practice such as the histological results, HIV-status, concurrent or previous conditions of relevance (e.g. TB) and the findings of the clinical evaluation.

**FIGURE 3:**
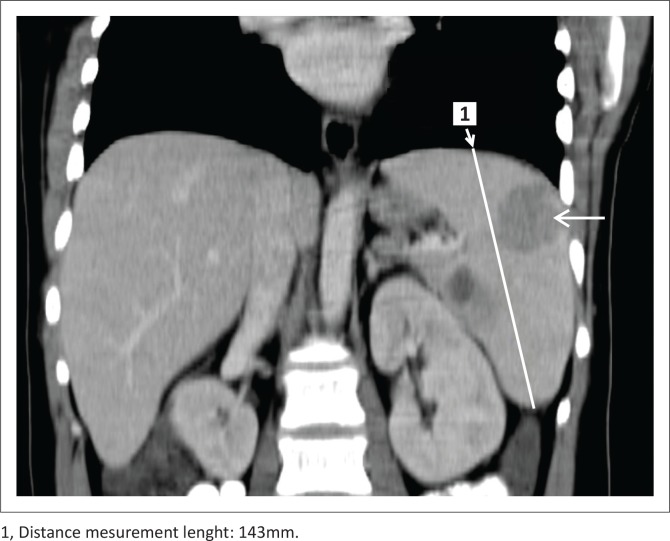
Splenomegaly with the spleen measuring > 13 cm in the craniocaudal dimension. Note the splenic lesion due to lymphoma (arrow).

The reviewers were then requested to complete data sheets for the baseline and restaging CT-scans according to the LC, which is elucidated in Figure 1. At baseline, the initial step was to identify and measure the target lesions and determine the sum of the product of the diameters (SPD). Each reviewer selected their own target lesions. The next step was to measure the craniocaudal length of the spleen on the coronal view, from its dome to its inferior tip (Figure 3). The final step was to identify and describe non-target disease, if any.

With the restaging CT-scans, the reviewers had to analyse the restaging scan and compare it with the previous scans. The previously identified target lesions were remeasured, and the new SPD and the percentage change from the baseline scan were calculated. The spleen was remeasured and compared to the previous results. With the change in the SPD and splenic dimension, the analysed variable was binary in nature, that is, ≥ 50% or < 50% for the SPD or ≤ or > 13 cm for the spleen. Next, previously identified non-target disease was reassessed as worse, stable or smaller, or resolved. If any new lesions attributable to lymphoma were detected, it was described as such. In case of progression, the product of the diameters of a single lesion was calculated, and the change from the nadir determined.

**FIGURE 4:**
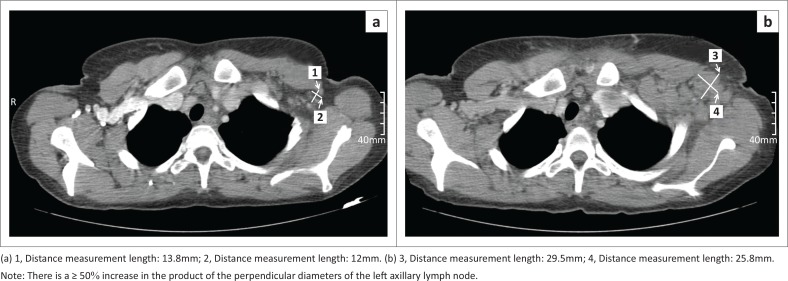
Progressive disease in Hodgkin’s lymphoma, initial computed tomography (CT) (a) and restaging CT (b).

**FIGURE 5:**
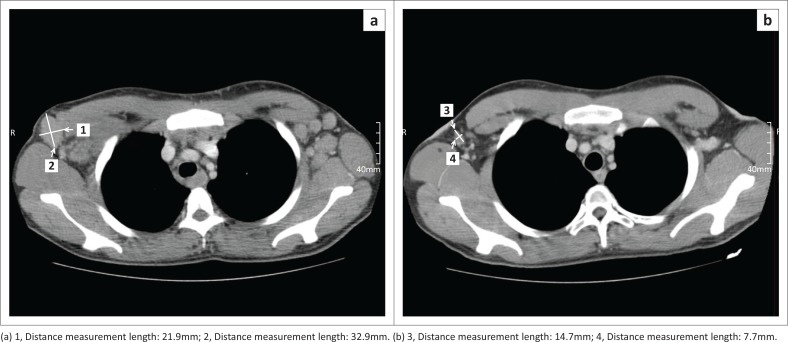
Hodgkin’s lymphoma, with the initial computed tomography (CT) (a) and restaging CT (b) Complete response to therapy with the axillary nodes regressing to normal size.

Finally, a response was assigned according to the LC (Figure 2), as CR, PR, SD or PD (Figures 4 and 5). Results were summarised by frequencies and percentages, with 95% confidence intervals for main outcomes. Kappa values were calculated to determine agreement between pairs of observers for the initial and follow-up restaging response classification.

### Ethical considerations

Ethical approval for the study was obtained from the Health Sciences Research Ethics Committee Administration (UFS-HSD 2017/1118) and Free State Department of Health (FS_201710_009). No patient consent was necessary as the study was a retrospective analysis of data with no alteration in medical management. Patient data could only be accessed by authorised medical personnel and were kept confidential at all times. Patient data were anonymised prior to data interpretation.

## Results

Patient age, lymphoma histology and treatment regimens are shown in Table 1. Ages ranged from 18–91 years, with a median of 38 years. Hodgkin’s lymphoma (nine cases) and diffuse large B-cell lymphoma (five cases) were the most common histological type. Adriamycin, bleomycin, vinblastine and dacarbazine (ABVD) and infusional cyclophosphamide, doxorubicin and etoposide (CDE) were the most common and second most common chemotherapy regimens respectively. Out of 21 patients, 14 were living with HIV.

**TABLE 1:** Patient histology, chemotherapy regimen and age.

Patient	Histology	Chemotherapy regimen	Age
1	DLBCL	R-CHOP	35
2	Plasmablastic lymphoma	CDE	46
3	Plasmablastic lymphoma	CDE	38
4	Plasmablastic lymphoma	CDE	25
5	HL	ABVD	26
6	HL	ABVD	45
7	DLBCL	CDE	49
8	HL	ABVD	19
9	Extra-nodal T-cell lymphoma	CHOP	41
10	DLBCL	Prednisone	91
11	Plasmablastic lymphoma	CHOP	40
12	Burkitt lymphoma	R-EPOCH	31
13	HL	ABVD	38
14	HL	ABVD	49
15	DLBCL	CDE	39
16	HL	ABVD	18
17	HL	ABVD	32
18	HL	ABVD	23
19	DLBCL	R-CDE	35
20	HL	Gemcitabine and/or Dexamethasone	56
21	Burkitt lymphoma	R-EPOCH	30

DLBCL, Diffuse large B-cell lymphoma; HL, Hodgkin’s lymphoma; R-CHOP, rituximab, cyclophosphamide, doxorubicin, vincristine and prednisone; CHOP, as above, rituximab omitted; CDE, cyclophosphamide, doxorubicin and etoposide (infusional regime); R-CDE, as above, rituximab added; ABVD, adriamycin, bleomycin, vinblastine and dacarbazine; R-EPOCH, rituximab, etoposide, prednisone, vincristine, cyclophosphamide and doxorubicin.

The frequency of inter-observer agreement was determined for each step in the process as previously set out (Figure 1) with the calculated percentages shown in Table 2. The *κ*-values for the initial restaging response assessment between pairs of observers ranged from 0.39–0.72 (moderate agreement); the follow-up restaging scans were analysed as a group with *κ*-values of 0.61–0.93 (moderate to strong agreement) between pairs of observers.

**TABLE 2:** Inter-observer agreement with staging and restaging.

Variable Steps in the staging and restaging process		Inter-observer agreement (%)
1. Baseline	Spleen size	100
	Absence or presence of non-target disease	76
2. Restaging (initial)	Change in the SPD	86
	Spleen size	90
	Re-evaluation of non-target disease	60
	Assessment of new disease	90
	Response classification	62^†^
3. Restaging (follow-up)	Change in the SPD	89
	Change in the PPD (with progression)	2/2^‡^
	Spleen size	95
	Re-evaluation of non-target disease	47
	Assessment of new disease	84
	Response classification	68^§^

SPD, sum of the product of the diameters; PPD, product of the perpendicular diameters.

† 95% confidence interval: 34% – 78%;

‡ this finding was only seen in two patients;

§ 95% confidence interval: 43% – 87%.

Finally, the overall concordance with regard to the combined initial and follow-up restaging response assessment was determined. Complete agreement during the whole cycle was seen in 11 out of 21 patients (52%; 95% confidence interval: 30% – 74%). The reasons for discordance in the remainder of the patients are summarised in Table 3. A significant change in management was possible in 8 out of 10 patients where dissent in the response assessment was demonstrated.

**TABLE 3:** Reasons for discordance with response assessment.

Reasons for discordance	Patient									
	1	3	4	6	7	9	11	12	15	20
Decrease in SPD of target lesions (< 50% vs. ≥ 50%)	†	‡	†	‡	†	‡	‡	‡	‡	‡
Assessment of target lesion regression to normal	‡	†	‡	‡	‡	‡	†	†	†	-
Assessment of spleen size regression to normal	‡	‡	‡	†	‡	‡	‡	‡	‡	‡
Identification and reassessment of non-target disease	‡	‡	‡	‡	‡	‡	†	‡	‡	‡
Identification of new lesions attributable to lymphoma	‡	‡	‡	‡	†	†	‡	‡	‡	†

† ‘reason for discordance’ was present in that specific patient;

‡ ‘reason for discordance’ was absent in that specific patient.

SPD, sum of the product of the diameters; vs., versus.

## Discussion

**FIGURE 6:**
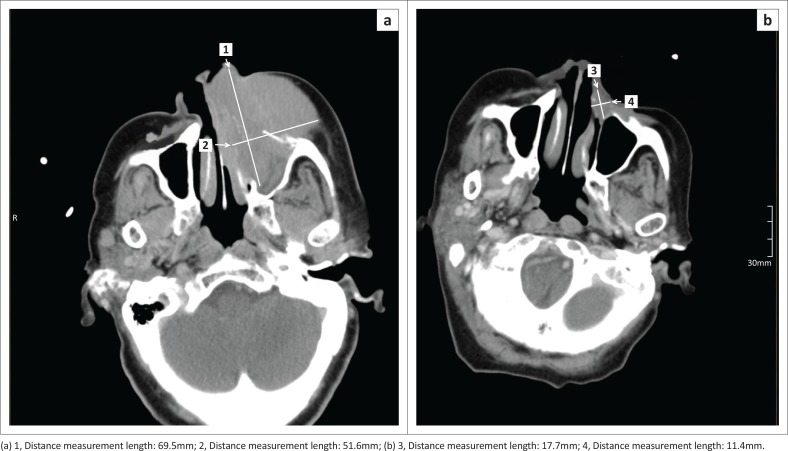
Plasmablastic lymphoma presenting as a maxillary mass. Initial computed tomography (CT) (a) and restaging CT (b), demonstrating a partial response.

The relatively high incidence (almost 20% of the total) of the otherwise rare plasmablastic lymphoma (Figure 6) in this study can be ascribed to the presence of HIV and oncogenic virus infection, such as Epstein–Barr and Kaposi sarcoma herpes virus, as discussed by Cesarman.^21^ Non-Hodgkin’s lymphomas are considered as an acquired immune deficiency syndrome (AIDS)-defining condition and related to persistent antigenic stimulation, immune suppression and genetic disruptions. Hodgkin’s lymphoma also has a higher incidence in patients with AIDS.

The high incidence of HIV (67%) corresponds with previously published data in the South African context, where de Witt et al.^22^ found that 80% of diffuse large B-cell lymphomas were HIV related. The presence of HIV-related lymphoma at a younger age and in advanced stages tends to be more aggressive and has a poorer prognosis overall. There are also other issues which complicate treatment, such as the higher prevalence of opportunistic infections and organ dysfunction for example bone marrow suppression and renal impairment. Simultaneous treatment with retroviral therapy when administering chemotherapy improves the response of the lymphoma.^9,21,23^

It is important to note that lymph node enlargement or splenomegaly does not necessarily equate to active lymphoma, especially in patients living with AIDS. HIV-associated lymphadenopathy in patients with active viral replication may be metabolically active with PET-CT, as will lymphoma.^5^ Assigning lung infiltrates, consolidation or nodules as either due to infection or lymphoma is particularly problematic in the local patient population, given the high burden of HIV and TB.

**FIGURE 7:**
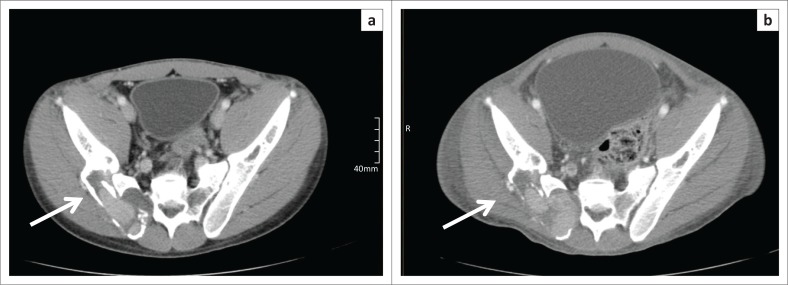
Restaging CT (a) Initial computed tomography (CT) (b) Right iliac skeletal lesion with a soft tissue component (arrow) where the soft tissue lesion proved difficult to measure reproducibly, resulting in significant inter-observer variability.

The most common reason (in 4 out of 10 patients) for response assessment discordance was an inter-observer difference in evaluation of target lesion regression to normal. This occurred most frequently with bony lesions with a soft tissue component where it was difficult to measure the soft tissue component reproducibly (Figure 7). It was also seen with irregular and ill-defined lymph node masses and with lymph nodes where the long-axis was not orientated parallel to the axial plane.

Other common sources of discrepancy were defining the decrease in the SPD as either^3^ ≥ 50% or < 50% and categorising new lesions as due to lymphoma or other causes (two and two patients, respectively, one with both). The former mainly occurred as a result of variability in the initial choice of target lesions. The latter was seen with lung lesions, where some reviewers regarded it as evidence of new or progressive lymphoma and others ascribed it to infectious or non-lymphoma-related causes. Reassessment of spleen size and non-target disease led to variance in response classification in two patients. There are definite limitations to the assessment of tumour involvement when using CT in lymphoma. A residual post-treatment mass can represent fibrosis instead of an active tumour. Lymphoma can also manifest without readily definable mass lesions, such as diffuse infiltration of the viscera or bone marrow.^12^

To the best of the authors’ knowledge, no published research specifically addresses inter-observer variability when applying the LC with CT in lymphoma. Therefore, we made a comparison with studies where observer variability was described with different response criteria, imaging modalities or as applied to cancers other than lymphoma. Obviously, response classification has to be precise and reproducible in order to guide clinical management and determine trial outcomes. Skougaard et al.^16^ assessed the inter-observer consistency (among 17 reviewers) of RECIST application with CT in a phase II trial in colon cancer and found that the overall response was differently classified in 17 out of 100 patients, although the change was potentially treatment altering in only six patients. Sources of discrepancy were the incorrect use of RECIST in the selection of target lesions, measurement of the tumour burden and identifying new lesions. The latter two discrepancies were also experienced in our study. The resultant variance in response classification was potentially treatment altering in 38% of our patients overall, which was higher than that experienced with the aforementioned study, where it was 6%.

Muenzel et al.^18^ studied intra- and inter-observer variability between four reviewers in the measurement of target lesions and its implication for response evaluation, specifically when applying the RECIST criteria with CT in 20 patients with various cancers. It was found that there was high variability in the sum of the measurements that potentially influenced the response classification, leading the researchers to suggest use of the mean results of all observers to improve consistency. Such an approach; however, would not be practical in the local setting, given the limited resources and personnel.

Interestingly, the aforementioned authors^18^ used proprietary software (Lesion Management Solutions) to facilitate computer-aided measurement of target lesions. Although the incorporation of computer-aided detection did not improve accuracy in their study, it led to significant timesaving, which should encourage radiologists to implement standardised restaging systems. Other investigators have shown that computer-aided volumetric tumour assessment is a promising technique in improving inter-observer variability, both with detection and diagnosis of lesions.^24,25,26^

Han et al.^19^ performed a retrospective review of 112 PET-CT-scans in 59 patients, using both the LC and the previous IHP criteria for response assessment in lymphoma, and found strong inter-observer agreement for initial restaging response assessment between the two readers (Cohen *κ* = 0.76) as applied to the IHP criteria but only moderate agreement with the LC (Cohen *κ* = 0.43). Most of the variability arose from an inconsistent interpretation of the residual fluorodeoxyglucose uptake. Moreover, our study revealed only moderate inter-observer agreement when using the LC, albeit with a different imaging modality.

Intra- and inter-observer variability of CT measurements in oncology was studied by McErlean et al.^13^ A total of 17 radiologists with varying experience measured lymph nodes, pulmonary and hepatic lesions in 205 patients with various cancers at different time points. Factors that positively correlated with measurement reproducibility were pulmonary location, smooth margins, larger lesion size and reader’s experience. Irregular, ill-defined lesions also decreased measurement reproducibility in our experience.

Accurate determination of PD is of particular importance, as progression-free survival is often utilised as a surrogate endpoint in cancer trials. Yoon et al.^20^ performed a meta-analysis to determine observer variability in the measurement of tumour burden with CT according to the RECIST criteria. Determining the overall tumour burden and the interval change in response to treatment varied to such an extent that misclassification as PD was observed. Variability decreased with an increase in the number of measured lesions. Our own experience mirrors this, where 14% of patients were inconsistently classified as having PD.

## Limitations

The study had a number of limitations that may have been a source of bias. Firstly, although all 21 patients had at least a baseline and initial restaging scan, seven patients’ subsequent follow-up restaging scans could not be included for analysis, because of the time constraints inherent to the study. Secondly, the study included a relatively small number of patients and reviewers, because of the mentioned time limitations. Despite these limitations; however, we regard the core conclusion of this research as valid, which is that there is significant inter-observer variability when applying the LC.

Thirdly, the reviewers reviewed the sequential scans in any given patient themselves. In routine daily practice, the baseline and restaging studies are most often reviewed by different readers, which can be expected to lead to even greater variability. In the fourth instance, there was a difference in the training level of the reviewers, varying from registrar to consultant level. However, no demonstrable trend, where any single reviewer was consistently at odds with the consensus, could be identified.

## Conclusion

This study demonstrated only moderate inter-observer agreement for response assignment when restaging lymphoma with computed tomography according to the LC. In at least one-third of patients, this would have led to a change in the treatment plan. These include switching to more toxic second-line chemotherapy, extended treatment with additional cycles of chemotherapy and invasive tissue biopsies.

Positron emission tomography-computed tomography has been established as the superior imaging modality when it comes to staging and restaging of most lymphomas. However, the reality in the developing world is that access to PET-CT is limited to academic referral centres in the largest metropolitan areas. Outside of these areas, the majority of patients are staged with CT only.

In the interest of consistency, and in keeping with the international guidelines, it is advisable for radiology departments to implement the LC when staging and restaging lymphoma. In our experience; however, there is a substantial risk of inter-observer variability with regard to response classification, which will influence patient management. Therefore, dedicated training is advised prior to introducing the LC in daily practice. Then, regular review and combined radiology–oncology meetings are recommended for quality control purposes. Also, when a change of therapy is being contemplated based on the imaging findings or when the imaging findings are at odds with the clinical response, histological confirmation should be sought.
